# Lipid Profiles and Fatty Acid Positional Distribution in Two Farmed Seahorse Species by Untargeted Lipidomics and Enzymatic Hydrolysis

**DOI:** 10.3390/biology15060495

**Published:** 2026-03-20

**Authors:** Tianxi Bi, Dandan Wang, Xiaoming Jiang, Tingting Lin, Yi Shao, Yuming Wang, Taher Abdelnaby, Lu Zhang, Chengcheng Wang, Tiantian Zhang

**Affiliations:** 1 SKL of Marine Food Processing & Safety Control, College of Food Science and Engineering, Ocean University of China, No.1299 Sansha Road, Qingdao 266404, China; 17860768561@163.com (T.B.); wdd2279371693@163.com (D.W.); jxm@ouc.edu.cn (X.J.); wangyuming@ouc.edu.cn (Y.W.); 2 Sanya Institute of Oceanography, Ocean University of China, Sanya 572000, China; 3 East China Sea Fisheries Research Institute, Chinese Academy of Fishery Sciences, Shanghai 200090, China; lintt@ecsf.ac.cn; 4 China National Center for Food Safety Risk Assessment (CFSA), Beijing 100022, China; shaoyi@cfsa.net.cn; 5 Food Science and Technology Department, Faculty of Agriculture, Al-Azhar University, Cairo 11651, Egypt; taherazher8@gmail.com; 6 National R&D Center for Freshwater Fish Processing, College of Life Science, Jiangxi Normal University, Nanchang 330022, China; zhanglu00104@163.com

**Keywords:** farmed seahorse, lipid profile, untargeted lipidomics, fatty acid positional distribution, *Hippocampus abdominalis*

## Abstract

Seahorses exhibit significant nutritional functions, including antioxidant and anti-tumor properties, with species like *Hippocampus abdominalis* and *Hippocampus erectus* now commercially farmed on a large scale. Notably, lipids are the key bioactive components distinguishing marine from terrestrial organisms. However, the lipidomic profiles of these cultured species are rarely reported, limiting their targeted nutritional application. Therefore, this study aimed to characterize and compare the lipid profiles of the newly introduced *H. abdominalis* with the currently farmed *H. erectus*. Methodologically, we employed an integrated approach utilizing untargeted lipidomics coupled with enzymatic hydrolysis to evaluate the lipid content, composition, molecular species, and fatty acid positional distribution of both species. The results revealed that triglyceride (TG) was the dominant lipid class in both seahorses. Notably, *H. abdominalis* exhibited higher overall phospholipid (PL) levels, along with elevated docosahexaenoic acid (DHA) and eicosapentaenoic acid (EPA) in its TG fraction. Positional distribution analysis demonstrated that DHA was evenly distributed at the *sn-1,3* and *sn-2* positions in *H. abdominalis*, but concentrated at the *sn-1,3* positions in *H. erectus*, whereas EPA was primarily localized at *sn-1,3* in both species. Furthermore, *H. abdominalis* contained higher DHA levels in phosphatidylcholine (PC) and phosphatidylethanolamine (PE) (predominantly at the *sn-1* position) and possessed abundant alkoxy ether PLs. Ultimately, this work fills crucial research gaps regarding the lipid characteristics of commercially cultured seahorses, providing a fundamental basis for species identification, nutritional evaluation, and the future development of seahorse-derived functional lipids.

## Simple Summary

Seahorses, such as commercially farmed *Hippocampus abdominalis* and *H. erectus*, possess notable nutritional properties including antioxidant and anti-tumor activities. Lipids are key bioactive components distinguishing marine organisms from terrestrial counterparts, yet the lipidomic profiles of cultured seahorses remain poorly characterized, limiting their targeted nutritional application. Here, we employed untargeted lipidomics combined with enzymatic hydrolysis to comprehensively compare the lipid profiles of these two species. Triglycerides (TG) were the dominant lipid class in both seahorses. *H. abdominalis* exhibited higher total phospholipids, as well as more unsaturated fatty acids in its TG fraction. Docosahexaenoic acid (DHA) was evenly distributed across sn-positions in *H. abdominalis* but concentrated at *sn-1,3* in *H. erectus*, while EPA was localized at *sn-1,3* in both. These findings support the health food industry in developing targeted nutritional supplements from sustainably farmed seahorses, and further tap into the health value of seahorses for human wellness.

## 1. Introduction

Seahorses are rare marine bony fishes with substantial culinary and medicinal potential [1,2]. Currently, seahorses in major Asian regions are primarily traded as traditional medicinal materials, while a small fraction are marketed as ornamental marine fishes [3]. Accordingly, aquaculture has emerged as a crucial solution to meet the edible and medicinal demand for seahorses [4]. Notably, *Hippocampus abdominalis* and *H. erectus* have been commercially cultivated on a global scale [5,6].

Numerous studies have demonstrated that seahorses not only contain beneficial nutrients such as proteins and minerals [7,8] but also rich in unsaturated fatty acids [9,10], including omega-3 long-chain unsaturated fatty acids such as docosahexaenoic acid (DHA, 22:6n-3) and eicosapentaenoic acid (EPA, 20:5n-3) [11]. These compounds exhibit significant biological activities in combating cardiovascular and cerebrovascular diseases as well as neurodegenerative disorders [12]. To date, there have been literature reports on the lipid profiles of *H. erectus* [13], while no research in the literature has yet reported the lipid profiles of *H. abdominalis* being successfully cultivated on a large scale. Thus, the analysis of *H. abdominalis* lipid profiles and the comparison with those of other seahorse species have become an urgent issue to address.

Notably, current lipid analysis of seahorse has primarily focused on lipid profiling, with few studies investigating the fatty acid positional distribution of their major molecular species. Importantly, the positional structure of triglyceride (TG) and phospholipid (PL) can affect the in vivo absorption and metabolism of lipids, which in turn influences their biological activity and function [14,15]. Furthermore, the enzymatic method is the most effective and rapid approach for identifying the structure of common lipid molecules [16]. Thus, identifying the fatty acid positional distribution of major lipids in seahorses is also a key topic in seahorse lipid analysis.

In this study, an analytical strategy integrating untargeted lipidomics and immobilized enzyme hydrolysis was used to systematically characterize the lipid profiles and the fatty acid positional distribution of major lipid molecular species in two major cultured seahorse species, *Hippocampus abdominalis* and *Hippocampus erectus*. By examining interspecific differences in lipid content, composition, and structure, this research fills a vital knowledge gap on seahorse nutrition and enables a deeper understanding of their health functions and reliable lipid profiling.

## 2. Materials and Methods

### 2.1. Experimental Sample

Two seahorse species, *H. abdominalis* and *H. erectus*, were donated by Weihai Yinze Biotechnology Co., Ltd. (Weihai, China). This facility is licensed for large-scale artificial seahorse breeding and strictly complies with the Convention on International Trade in Endangered Species of Wild Fauna and Flora (CITES) and national aquatic wildlife protection laws. The seahorses used in our research are in full compliance with the basic principles specified for seahorses intended for scientific research purposes under CITES Appendix II. Rearing conditions for both species were strictly standardized and monitored, including dissolved gas concentrations, light intensity, water temperature, and tank hygiene. The juveniles were fed *Artemia nauplii*, while adults were given a diet of opossum shrimp. Upon maturity, 100 individuals of each species (1:1 male-to-female ratio, which is consistent with the roughly equal proportion of males to females in consumption, as well as the typical ratio observed under aquaculture conditions) were randomly sampled from separate same-batch culture tanks. Following guidelines approved by the Institutional Animal Care and Use Committee (IACUC), seahorses were humanely euthanized via overdose anesthesia with tricaine methanesulfonate (MS-222). Euthanized specimens were rinsed thoroughly with 0.22 μm-filtered seawater to remove surface mucus and exogenous impurities, then blotted dry. Each seahorse species was further assigned to three subgroups and subjected to frozen transportation at –20 °C to the laboratory. Subsequently, the samples were freeze-dried with a Scientz-18N freeze dryer (Ningbo Xinzhi Co., Ltd., Ningbo, China), ground into a fine powder, and processed for subsequent lipid extraction and analysis.

### 2.2. Reagents

Chloroform, methanol, hydrochloric acid, and sodium hydroxide used in this study were all of reagent grade and purchased from Sinopharm Chemical Reagent Co., Ltd. (Shanghai, China). n-Hexane, isopropanol, acetonitrile, and ammonium formate were of chromatographic grade and purchased from Sigma-Aldrich (St. Louis, MO, USA). Thin-layer chromatography (TLC) silica gel plates were provided by Yantai Huayang New Material Technology Co., Ltd. (Yantai, China), and the triglyceride (TG) assay kit (Cat. No.: A020-1-1) was purchased from Nanjing Jiancheng Bioengineering Institute (Nanjing, China).

### 2.3. Lipid Extraction and Purification

The extraction of total lipids from seahorses was performed with appropriate modifications based on the Folch method [17]. Briefly, a specified mass of dried *H. abdominalis* and *H. erectus* powder was mixed with 10 volumes (*m*/*v*) of extraction solvent (chloroform: methanol = 2:1, *v*/*v*), respectively. Butylated hydroxyanisole (BHA) was added to the chloroform–methanol extraction solution at a concentration of 100 μg/mL to prevent lipid oxidation. After multiple processing steps, the lower organic phase was collected and concentrated under reduced pressure to remove organic solvents, yielding total seahorse lipids. Subsequently, column chromatography was performed to isolate the triglyceride (TG), phosphatidylcholine (PC), and phosphatidylethanolamine (PE) fractions. Specifically, 10 g of total lipids were loaded onto a silica gel column (packed with 60 g of silica gel; internal diameter = 2 cm). Pure dichloromethane was initially adopted as the eluent, and triglycerides eluted at the third column volume (CV). After the complete collection of all neutral lipid fractions, phospholipid components were eluted using a dichloromethane/methanol mixture (5:1, *v*/*v*), from which phosphatidylethanolamine (PE) and phosphatidylcholine (PC) were sequentially isolated.

The eluted fractions were subsequently identified and their purities were initially evaluated via thin-layer chromatography (TLC). The analysis was performed on silica gel G plates with dimensions of 10 × 5 cm, a sorbent particle size of 5–20 μm, and a stationary phase layer thickness of 0.23 mm. Approximately 100 μg of the lipid sample was applied. For neutral lipids (TG), the mobile phase consisted of petroleum ether/diethyl ether/acetic acid (85:15:1, *v*/*v*/*v*). For polar lipids (PC and PE), the mobile phase was chloroform/methanol/water (65:25:4, *v*/*v*/*v*). Lipid-containing bands were visualized utilizing the iodine vapor method, and their identities were confirmed by comparing them with commercial standards, exhibiting Rf values of 0.87 for TG, 0.56 for PC, and 0.84 for PE.

Subsequently, the purity of the collected lipid fractions (PC, PE, and TG) was strictly quantified using a Waters HPLC system coupled with a Waters 2424 Evaporative Light Scattering Detector (ELSD) (Waters Corporation, Milford, MA, USA). Separation was achieved on a YMC-Did-120 silica-bonded diol column (250 × 4.6 mm, 5 μm sorbent particle size) maintained at 55 °C. The mobile phase system consisted of phase A (n-hexane/isopropanol/acetic acid/triethylamine = 81.42:17:1.5:0.08, *v*/*v*/*v*/*v*) and phase B (isopropanol/water/acetic acid/triethylamine = 84.42:14:1.5:0.08, *v*/*v*/*v*/*v*) at a flow rate of 1.0 mL/min. A gradient elution program was applied as follows: 0 min, 95% A; 5 min, 80% A; 7 min, 60% A; 17 min, 30% A; 23 min, 0% A; 25.5 min, 95% A; and 30 min, 95% A. The ELSD conditions were optimized with a detector sensitivity gain value of 50, a drift tube temperature of 50 °C, and a nebulizer gas pressure of 25 psi, operating in heating mode (power level 60%). For each analysis, an injection volume of 20.0 μL of the lipid fraction at a concentration of 1.0 mg/mL was introduced into the system.

The purities of all the aforementioned substances, as determined by ELSD peak area normalization, were confirmed to be above 90%. These highly pure samples were then frozen and stored at −20 °C for subsequent analysis.

### 2.4. Analysis of Fatty Acid Compositions

TG were quantified using a commercial assay kit (Cat. No. A020-1-1, Nanjing Jiancheng Bioengineering Institute). Phospholipids (PL) were determined via the molybdenum blue colorimetric method [18]. Briefly, sample solutions were prepared with chloroform–methanol (2:1, *v*/*v*) as the solvent, followed by digestion with perchloric acid. The inorganic phosphorus content in the samples was then measured, and phospholipid content was calculated by multiplying the measured inorganic phosphorus content by a conversion factor of 25.

The total lipids, as well as the samples separated by silica gel column chromatography, were individually placed into methylation reagents (hydrochloric acid: methanol = 1:5, *v*/*v*), homogenized, and reacted in a metal bath at 90 °C for 3 h, with homogenization performed every 30 min during the reaction. After cooling, n-hexane was added for extraction. Once the mixture was allowed to stand and separate into layers, 1 mL of the supernatant was collected and dried by nitrogen blowing. The dried sample was then re-dissolved in 50 μL of n-hexane and used for subsequent gas chromatography analysis [19].

The GC system was set as follows: An Agilent 7820A Gas Chromatograph (Agilent Technologies, Santa Clara, CA, USA) with a capillary column (AE. FFAP; 30 m × 0.32 mm × 0.25 μm film thickness); The gas chromatography detector employed was a Flame Ionization Detector (FID). The detector and injector temperatures were maintained at 250 °C and 240 °C, respectively. The column temperature program was programmed as follows: initial temperature of 170 °C, ramped up to 240 °C at a rate of 3 °C/min, and then held isothermally at 240 °C for 35 min. High-purity nitrogen was used as the carrier gas, with a constant flow rate of 1.0 mL/min. The total runtime for a single sample analysis was 48.5 min [20].

### 2.5. Ultra-High-Performance Liquid Chromatography-Tandem Mass Spectrometry (UPLC-MS) Analysis

For lipid profile analysis, a high-resolution orbitrap mass spectrometer (UPLC-Q-Orbitrap) coupled with an ACQUITY UPLC BEH C18 column (2.1 mm × 100 mm, 1.7 μm) was used. The system included a UPLC module from Waters Corporation (Milford, MA, USA) and an electrospray ionization (ESI) source from Thermo Fisher Scientific (Waltham, MA, USA). Mobile phase A consisted of acetonitrile/water (60:40, *v*/*v*) containing 10 mmol/L ammonium formate, and mobile phase B was isopropanol/acetonitrile (90:10, *v*/*v*). The gradient elution program was as follows: initial conditions (100% A); linear increase to 32% B over 1.5 min; further increase to 85% B by 15.5 min; rapid ramp to 97% B within 0.1 min, held for 2.4 min; then decreased to 32% B within 0.1 min, held for 1.9 min. Curtain Gas (Cur) was set to 35 psi; the flow rates of both Gas 1 (GS1) and Gas 2 (GS2) were configured to 55 psi; Ion Source Voltage/Float (ISVF) was adjusted to 5500/−4500 V; Ion Source Temperature (TEM) was maintained at 350 °C; Declustering Potential (DP) was set to 80 V; Entrance Potential (EP) was set to 10 V; and Collision Cell Exit Potential (CXP) was set to 15 V. Quantitative analysis of total ion current (TIC) chromatograms was performed using Lipid Search Launcher 4.2.21 software (Thermo Fisher Scientific) [21].

### 2.6. Determination of Fatty Acid Positional Distribution

To determine the positional distribution of fatty acids in TG, PC, and PE, 2 mg of the lipid sample and 1 mg of immobilized Novozym 435 lipase were placed into each centrifuge tube separately. The reaction was carried out in 40 mL of 95% ethanol [22]. After 12 h, the immobilized enzymes were removed from the enzymatic hydrolysates via centrifugation, and the enzyme-free mixture was heated at 95 °C for 1 min under a nitrogen atmosphere and immediately quenched in an ice bath. The mixture was then dried and concentrated in a nitrogen environment prior to high-performance thin-layer TLC separation.

The TG enzymatic hydrolysates were separated using a solvent system of petroleum ether, diethyl ether, and acetic acid at a volume ratio of 85:15:1 (*v*/*v*/*v*), from which the produced monoacylglycerols (MG, Rf = 0.25) and the unreacted initial TG (Rf = 0.87) were obtained. The PC and PE enzymatic hydrolysates were treated with a solvent system of chloroform, methanol, and water at a volume ratio of 65:25:4 (*v*/*v*/*v*), obtaining unreacted PC (Rf = 0.56) and PE (Rf = 0.84), along with their respective products, LPC (Rf = 0.27) and LPE (Rf = 0.33). Finally, the target bands were scraped off and subjected to fatty acid composition analysis.

### 2.7. Statistical Analysis

Triplicate experiments were conducted for both the quantitative analysis of lipid molecules and the determination of lipid class composition, while triplicate measurements were performed for fatty acid component analysis. All data in the figures are expressed as the mean ± standard error of the mean (SEM). Statistical analysis was performed using one-way analysis of variance (ANOVA), followed by Tukey’s post hoc test to identify significant differences between groups. Differences were considered statistically significant at *p* < 0.05 and marked with different letters. Orthogonal Partial Least Squares-Discriminant Analysis (OPLS-DA), heatmap analysis, and volcano plot generation were carried out using the free online data analysis platform Metware Cloud (https://cloud.metware.cn).

## 3. Results

### 3.1. Lipid Content and Lipid Class Composition

As shown in Supplementary Figure S1, the lipid extraction yield of *H. abdominalis* (76.4 mg/g dry weight) was higher than that of *H. erectus* (69.1 mg/g dry weight), indicating that *H. abdominalis* exhibited a higher lipid content. The fatty acid content of the total lipids of *Hippocampus abdominalis* and *Hippocampus erectus* were analyzed using gas chromatography (GC) through comparison with the mixed standard chromatogram (Figure S2), with the results summarized in Table 1. For *H. abdominalis*, 16 fatty acids with carbon chain lengths ranging from C14 to C22 were identified, with major components including C16:0, C16:1, C18:0, C18:1, C20:5n-3 (EPA), and C22:6n-3 (DHA). Saturated fatty acids (SFA) accounted for 38.9% of its total lipids (predominantly C16:0 at 23.1%, C18:0 at 9.6%, and C14:0 at 3.6%), while 10 unsaturated fatty acid (UFA) species contributed 61.1% (monounsaturated fatty acids (MUFA) at 29.9%, mainly C18:1 at 20.8% and C16:1 at 7.4%; polyunsaturated fatty acids (PUFA) at 31.3%, dominated by DHA at 14.37% and EPA at 8.3%. For *H. erectus*, the major fatty acids were C16:0 (27.4%), C18:1 (15.6%), C18:0 (11.0%), C16:1 (8.5%), and C14:0 (7.6%), with SFA, MUFA, and PUFA accounting for 50.4%, 24.1%, and 25.6% of total lipids, respectively. The UFA content of *H. abdominalis* (59.4%) was higher than that of *H. erectus* (49.6%), and the total DHA + EPA content of both species was higher than that of *H. kelloggi*.

### 3.2. Differences in TG Molecular Species Between Two Seahorse Species and Fatty Acid Positional Distribution in TG

**Figure 1:**
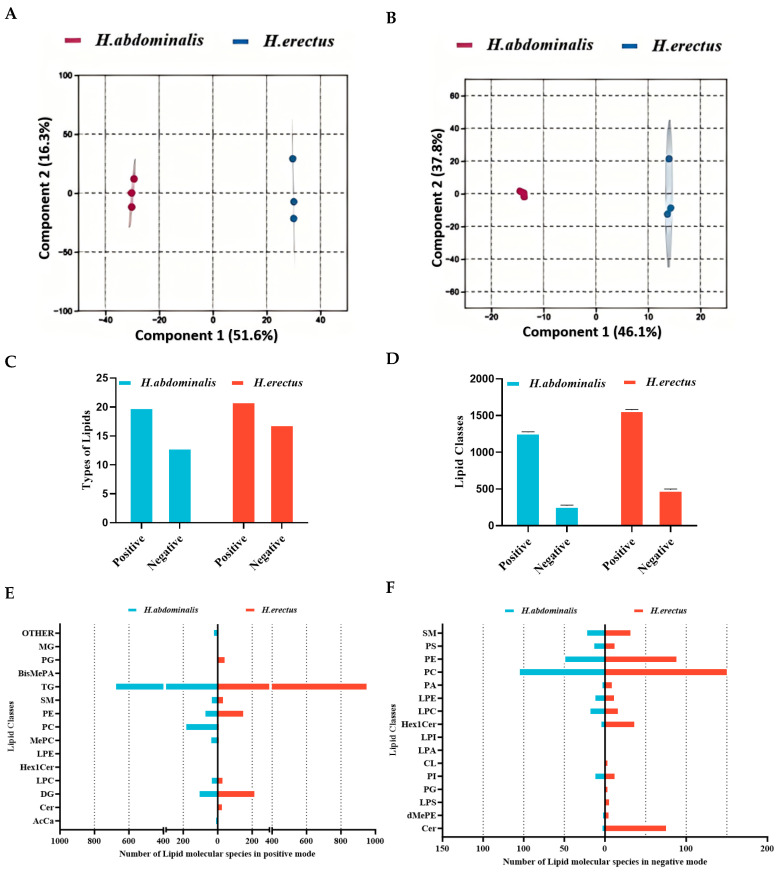
Orthogonal Partial Least Squares-Discriminant Analysis (OPLS-DA) of lipidomics data from *H. abdominalis* and *H. erectus* in positive mode (**A**) and negative mode (**B**); types of lipids (**C**) and number of lipid classes (**D**) of the two seahorse species under different ion modes; number of lipid molecules in different lipid classes in positive mode lipidomics (**E**) and negative mode lipidomics (**F**). BisMePA for Bis(methoxyethyl)phosphatidylethanolamine, Cer for Ceramide, CL for Cardiolipin, dMePE for Dimethylethanolamine Phosphatidylethanolamine, Hex1Cer for Monoglucosylceramide, LPA for Lysophosphatidic Acid, LPC for Lysophosphatidylcholine, LPE for Lysophosphatidylethanolamine, LPI for Lysophosphatidylinositol, LPS for Lipopolysaccharide, PA for Phosphatidic Acid, PI for Phosphatidylinositol, SM for Sphingomyelin.

According to Figure S3, TG was the major component of seahorse lipids, with *H. erectus* (26.7%) having a slightly lower TG content than *H. abdominalis* (29.5%). Phospholipids (PL) accounted for 9.2–12.3% of total lipids, with *H. abdominalis* showing a higher PL content than *H. erectus*. Then the lipid components of the two seahorse species were analyzed by means of lipidomics. Preliminary findings indicated that there were differences between them through comparison with the Total Ion Chromatogram (TIC) profiles under the two modes (Figures S4 and S5). OPLS-DA results (Figure 1A,B) revealed significant differences in lipid molecular characteristics between the two species. In positive ion mode, the number of lipid types was comparable between them, but *H. erectus* had more lipid types in negative ion mode. Regarding lipid class composition (Figure 1D–F), *H. erectus* exhibited a significantly higher diversity of lipid classes across both ion modes. Upon data integration, TG emerged as the predominant lipid class. Notably, several specific lipid classes, including LPA, LPI, CL, and BisMePA, were entirely absent in *H. abdominalis*.

**Figure 2:**
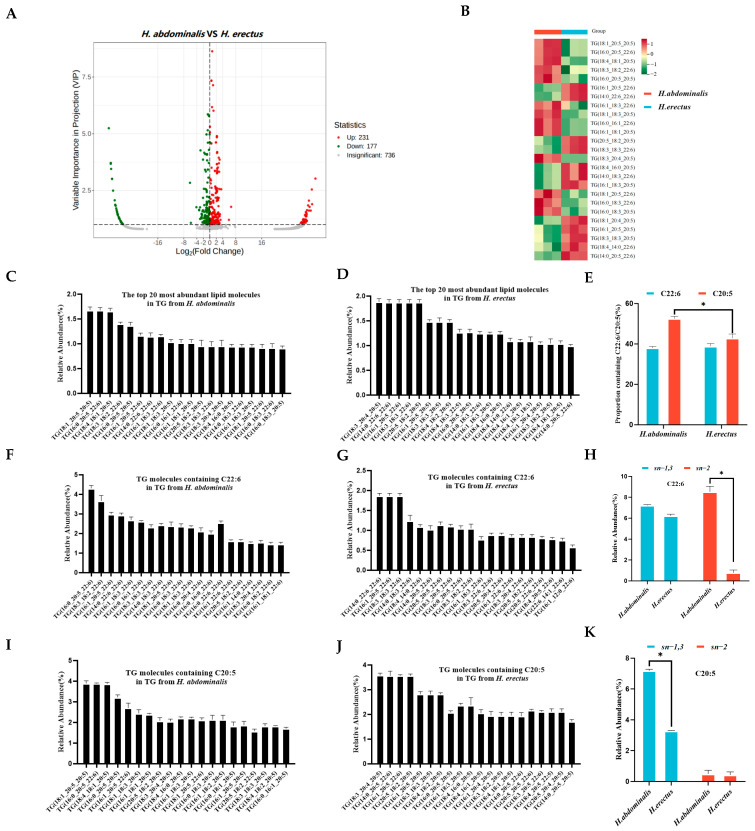
Analysis of differential triglycerides in *H. abdominalis* and *H. erectus*. The volcano plot (**A**). Differential lipid species were selected out by using the criteria of *p* Value |log_2_FoldChange| > 1, VIP score > 1 in the volcano plot (n = 3). The lipid thermograms (**B**) of 25 triglycerides in two kinds of *Hippocampus* were screened by relative content and significance. Relative abundances of the top 20 TG lipid molecular species in TG from *H. abdominalis* (**C**) and *H. erectus* (**D**) under positive mode; the ratio of the number of TG molecular species containing C22:6 and C20:5 from the two seahorse species (**E**); the relative abundance of top 20 lipid molecules containing C22:6 in TG from *H. abdominalis* (**F**) and *H. erectus* (**G**); fatty acid intra-positional composition of C22:6 in TG of the two seahorse species (**H**); the relative abundance of top 20 lipid molecules containing C20:5 in TG from *H. abdominalis* (**I**) and *H. erectus* (**J**); fatty acid intra-positional composition of C20:5 in TG of the two seahorse species (**K**). Values are expressed as mean ± standard error of the mean (SEM). The number of independent biological replicates is n = 3 for each group. * indicates a significant difference between the two groups (*p* < 0.05).

A total of 408 significantly different TG lipid species were identified between the two species (Figure 2A), with 231 upregulated and 177 downregulated in *H. abdominalis* relative to *H. erectus*. Differential TG species screened by |log_2_ fold change| > 1 and VIP score > 1 were visualized via volcano plots (Figure 2B), and 25 of the top 20 most abundant TG species in each seahorse showed significant interspecific differences. *H. abdominalis* had 683 detected TG molecular species, while *H. erectus* had 958 (Figure 1E). The most abundant TG species in both seahorses almost all contained C20:5 or C22:6 (Figure 2C,D). The proportion of C22:6-containing lipid species was approximately 38% in both, while higher levels of C22:6-containing lipids (e.g., TG(C16:0/C20:5/C20:6) at 4.3%) and slightly higher levels of C20:5-containing lipids (e.g., TG(C18:1/C20:5/C20:5) at 3.9%) than *H. erectus*. The proportion of C20:5-containing species was significantly higher in *H. abdominalis* (52.6%) than in *H. erectus* (42.9%) (Figure 2E). Furthermore, *H. abdominalis* contained a substantial amount of the lipid TG(C14:0/C22:6/C22:6) at 1.9%, and its dominant C20:5-containing lipid was TG(C18:3/C20:4/C20:5) at 3.6% (Figure 2F,G,I,J). Enzymatic hydrolysis results (Table S1, Figure 2H,K) showed that in *H. abdominalis*, C22:6 was distributed at both *sn-1,3* (7.2%) and *sn-2* (8.5%) positions of TG, while in *H. erectus*, C22:6 was predominantly at *sn-1,3* positions (6.2%). C20:5 was concentrated at *sn-1,3* positions in both, with a higher content in *H. abdominalis* (7.2%) than in *H. erectus* (3.3%).

### 3.3. Differences in PL Molecular Species Between Two Seahorse Species

**Figure 3:**
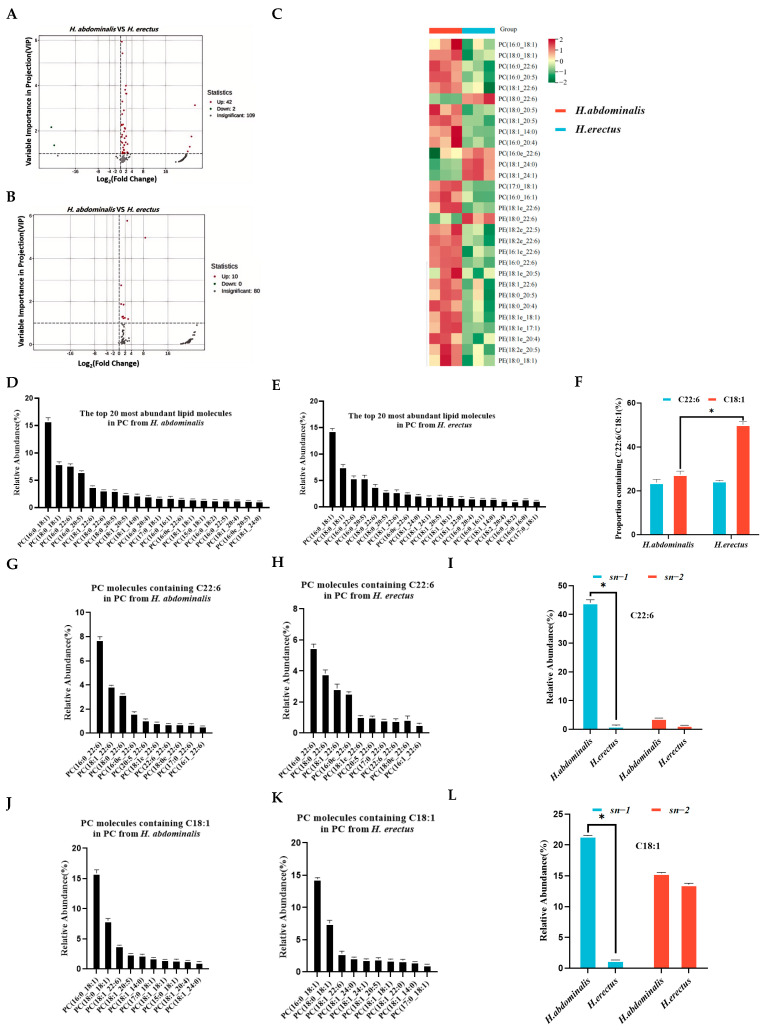
Analysis of differential PC (**A**) and PE (**B**) in *H. abdominalis* and *H. erectus*. Differential lipid species were selected out by using the criteria of *p* Value |log_2_FoldChange| > 1, VIP score > 1 in the volcano plot (n = 3). The lipid thermograms (**C**) of 15 kinds of PC and 15 kinds of PE in the two kinds of *Hippocampus* were screened by relative content and significance. Relative abundances of the top 20 PC lipid molecular species in PC from *H. abdominalis* (**D**) and *H. erectus* (**E**) under positive mode; the ratio of the number of PC molecular species containing C22:6 and C18:1 from the two seahorse species (**F**); the relative abundance of top 20 lipid molecules containing C22:6 in PC from *H. abdominalis* (**G**) and *H. erectus* (**H**); fatty acid intra-positional composition of C22:6 in PC of the two seahorse species (**I**); the relative abundance of top 20 lipid molecules containing C18:1 in PC from *H. abdominalis* (**J**) and *H. erectus* (**K**); fatty acid intra-positional composition of C18:1 in PC of the two seahorse species (**L**). Values are expressed as mean ± standard error of the mean (SEM). The number of independent biological replicates is n = 3 for each group. * indicates a significant difference between the two groups (*p* < 0.05).

For PL components, 44 differential PC species were identified between the two seahorses (Figure 3A), with 42 upregulated and 2 downregulated in *H. abdominalis*. Ten differential PE species were detected (Figure 3B), all upregulated in *H. abdominalis*. Volcano plots visualized these differential species (Figure 3C). Among the top 10 most abundant PC and PE species in each seahorse, 15 PC and 15 PE species showed significant interspecific differences, with most being more abundant in *H. abdominalis*. *H. abdominalis* had 51 PE molecular species, compared to 90 in *H. erectus* (Figure 1F).

### 3.4. Molecular Species and Positional Distribution of Fatty Acids in PC

The most abundant PC species in both seahorses almost all contained C22:6 or C18:1 (Figure 3D,E). The proportion of C22:6-containing PC species was slightly lower in *H. abdominalis* (23.8%) than in *H. erectus* (24.5%), while the proportion of C18:1-containing PC species was significantly lower in *H. abdominalis* (27.5%) than in *H. erectus* (50.2%) (Figure 3F). *H. abdominalis* had higher levels of C22:6-containing PC species (e.g., PC(C16:0/C22:6) at 7.8%) and slightly higher levels of C18:1-containing PC species than *H. erectus* (PC(C16:0/C22:6) at 5.5%) (Figure 3G,H,J,K). The most abundant PC species in both was PC(C16:0/C18:1), with relative contents of 15.9% (*H. abdominalis*) and 14.4% (*H. erectus*). Enzymatic hydrolysis results (Table S2, Figure 3I,L) showed that PC in *H. abdominalis* had high DHA content (44.0% at *sn-1* position), while DHA content in *H. erectus* was approximately 1% at both *sn-1* and *sn-2* positions. C18:1 in *H. abdominalis* was primarily at *sn-1* (21.4%), while in *H. erectus*, C18:1 content at *sn-1* (15.1%) and *sn-2* (13.6%) were similar.

### 3.5. Molecular Species and Positional Distribution of Fatty Acids in PE

**Figure 4:**
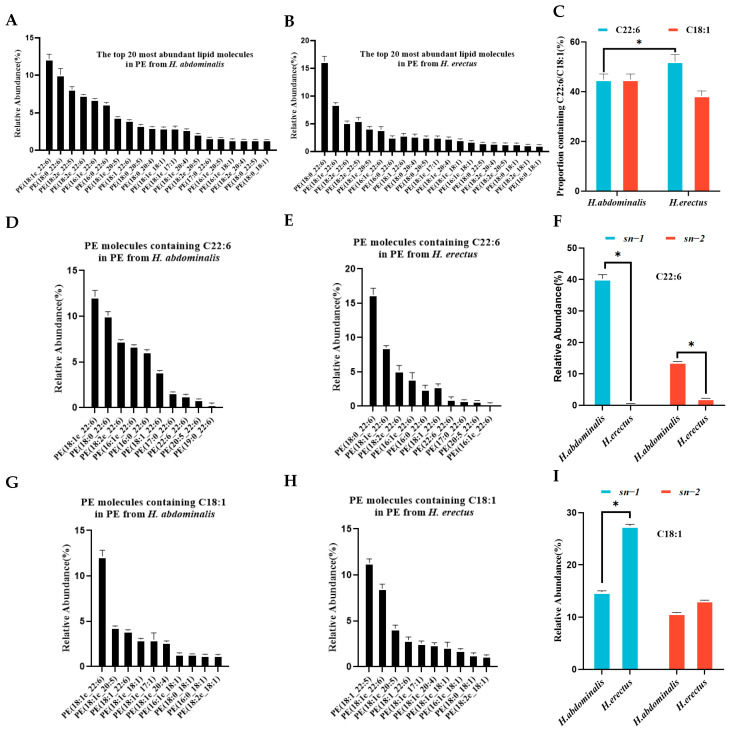
Relative abundances of the top 20 PE lipid molecular species in PE from *H. abdominalis* (**A**) and *H. erectus* (**B**) under positive mode; the ratio of the number of PE molecular species containing C22:6 and C18:1 from the two seahorse species; the ratio of the number of PE molecular species containing C22:6 and C18:1 from the two seahorse species (**C**); the relative abundance of top 20 lipid molecules containing C22:6 in PE from *H. abdominalis* (**D**) and *H. erectus* (**E**); fatty acid intra-positional composition of C22:6 in PE of the two seahorse species (**F**); the relative abundance of top 20 lipid molecules containing C18:1 in PE from *H. abdominalis* (**G**) and *H. erectus* (**H**); fatty acid intra-positional composition of C18:1 in PE of the two seahorse species (**I**). Values are expressed as mean ± standard error of the mean (SEM). The number of independent biological replicates is n = 3 for each group. * indicates a significant difference between the two groups (*p* < 0.05).

The most abundant PE species in both seahorses almost all contained C22:6 or C18:1 (Figure 4A,B). The proportion of C22:6-containing PE species was lower in *H. abdominalis* (44.9%) than in *H. erectus* (52.2%), while the proportion of C18:1-containing PE species was higher in *H. abdominalis* (44.9%) than in *H. erectus* (38.4%) (Figure 4C). *H. abdominalis* had a significantly higher abundance of alkoxy ether phospholipid PE, such as PE(C18:1e/C22:6) (12.2%), PE(C18:2e/C22:6) (7.4%), and PE(C18:1e/C20:5) (4.4%) (Figure 4D,E,G,H). Enzymatic hydrolysis results (Table S3, Figure 4F,I) showed that PE in *H. abdominalis* had high DHA content (40.2% at *sn-1*, 13.72% at *sn-2*), while DHA content in *H. erectus* was below 2.5% at *sn-2*. C18:1 in *H. abdominalis* was relatively evenly distributed between *sn-1* (14.8%) and *sn-2* (10.8%), while in *H. erectus*, C18:1 content was higher at *sn-1* (27.4%) than at *sn-2* (13.2%).

## 4. Discussion

The present study systematically characterized the lipid composition, molecular species, and fatty acid positional distribution of two seahorse species (*H. abdominalis* and *H. erectus*), revealing significant interspecific differences. Given that these species are now globally cultured under similar controlled conditions, these variations are not driven by their immediate environment, but rather can be attributed to species-specific metabolic characteristics [23,24,25]. These inherent differences highlight the superior nutritional value and functional potential of *H. abdominalis* compared to *H. erectus*. In terms of lipid class composition, both seahorse species showed TG as the predominant lipid class, which is consistent with the lipid profile of many deep-sea fish [26]. Notably, the two seahorse species were relatively abundant in the diversity of their DG molecular species, a phenomenon that might be associated with the hydrolysis of TG catalyzed by TG lipase within the organisms during transportation and storage. Furthermore, The higher UFA content (especially DHA and EPA) in *H. abdominalis* (61.1%) compared to *H. erectus* (49.6%) is a key finding, as PUFAs like DHA and EPA are well-documented for their beneficial effects on human health, including lowering blood lipids and alleviating atherosclerosis [27]. The total DHA + EPA content of both seahorses was higher than that of *H. kelloggi* [9], further confirming seahorses as a valuable source of these essential fatty acids.

The OPLS-DA results demonstrated distinct lipid molecular characteristics between the two species, with *H. erectus* possessing more lipid types, particularly in negative ion mode. This difference may be related to the ionization properties of lipids: triglycerides are effectively ionized in positive ion mode, while phospholipids (with negatively charged phosphate groups) form stable [M-H]^−^ or [M+HCOO]^−^ ions in ESI negative mode, facilitating their detection [28,29]. The higher relative proportion of PC in *H. abdominalis* (9.9% vs. 9.0% in *H. erectus*) is noteworthy, as PC is a critical component of cell membrane and plays important roles in lipid metabolism. The analysis of TG molecular species revealed 408 differential species between the two seahorses, with *H. abdominalis* showing higher levels of DHA- and EPA-containing lipids. The enrichment of DHA and EPA in the major TG species of both seahorses, even at moderate overall proportions in total lipids, indicates targeted accumulation of these PUFAs, which is consistent with findings in some marine fish species [30]. Furthermore, we focus primarily on the differences in fatty acids at the *sn-1,3* and *sn-2* positions of TG. The rationale behind this focus is that triglycerides in the digestive tract are preferentially hydrolyzed by pancreatic lipase into *sn-2* monoacylglycerol prior to absorption. The experimental results demonstrated that the positional distribution of fatty acids in TG further amplifies the nutritional superiority of *H. abdominalis* in that DHA is enriched at the *sn-2* position (8.5%), a position with higher fatty acid absorption efficiency while the EPA content at the *sn-1,3* positions is significantly higher than that in *H. erectus*. This distinct positional distribution pattern facilitates the more efficient exertion of DHA and EPA’s nutritional benefits [31].

For phospholipids, *H. abdominalis* showed upregulation of most differential PC and PE species, despite having fewer total PE molecular species than *H. erectus*. The high abundance of alkoxy ether phospholipid PE in *H. abdominalis* (e.g., PE(C18:1e/C22:6)) is particularly significant, as these lipids possess superior oxidation resistance and are associated with multiple nutritional function [32,33]. The positional distribution of fatty acids in PC and PE also contributes to the higher lipid absorption efficiency of *H. abdominalis* in that DHA is highly concentrated at the *sn-1* position of PC (44.0%) and PE (40.2%), while the even distribution of C18:1 in PE may further optimize lipid metabolism [34].

Collectively, these results indicate that *H. abdominalis* may possess superior nutritional value to *H. erectus* from the aspect of lipid and thus holds greater potential for the development of lipid-derived functional ingredients and precision nutrition products [35]. The distinct lipid profiles of the two seahorse species furnish a scientific foundation for their targeted application in the food and pharmaceutical industries. Given that the present study is constrained by the lack of quantitative analysis of lipid molecular species, future research could focus on conducting in-depth quantitative investigations to clarify the precise lipid composition of the two seahorse species, as well as evaluating the in vitro and in vivo biological activities of these target lipids.

## 5. Conclusions

This study clarified the lipid differences between two farmed seahorse species, *H. abdominalis* and *H. erectus*, by comparing their lipid content, composition, molecular species, and fatty acid positional distribution and using untargeted lipidomics combined with enzymatic hydrolysis. Of these lipids, triglyceride (TG) was the major lipid class in both species. Interestingly, *H. abdominalis* had higher levels of phospholipids (PL) than *H. erectus*. Specifically, the content of docosahexaenoic acid (DHA) and eicosapentaenoic acid (EPA) in the TG of *H. abdominalis* was higher than that in *H. erectus*. Moreover, DHA in *H. abdominalis* was evenly distributed at the *sn-1,3*, and *sn-2* positions, while in *H. erectus*, DHA was mainly distributed at the *sn-1,3* positions. In contrast, EPA was concentrated at the *sn-1,3* positions in both seahorse species. Additionally, the DHA content in phosphatidylcholine (PC) and phosphatidylethanolamine (PE) of *H. abdominalis* was much higher than that in *H. erectus*, and this DHA was mainly concentrated at the *sn-1* position. Notably, *H. abdominalis* also contained a large amount of alkoxy ether phospholipids. This study addresses the existing research gaps in the lipid profiles of two commercially viable large-scale cultured seahorse species. Specifically, it characterizes the interspecific differences in lipid molecular species, as well as the difference in fatty acids at distinct positions of the major molecular species (i.e., TG, PC, and PE). These findings provide a fundamental basis for the species identification and development of functional lipids derived from seahorses.

## Figures and Tables

**Table 1:** Relative abundance and content of fatty acids in two seahorse species.

Fatty Acids	*H. abdominalis*			*H. erectus*		
	Relative Abundance (%)	Contents (mg/g Total Lipids)	Contents (mg/g DW)	Contents (mg/g Total Lipids)	Relative Abundance (%)	Contents (mg/g DW)
C14:0	3.6 ± 0.0	18.2 ± 0.1	1.39 ± 0.01	7.6 ± 0.1	39.9 ± 1.9	2.76 ± 0.13
C16:0	23.1 ± 0.1	116.8 ± 0.4	8.92 ± 0.03	27.4 ± 0.1	159.6 ± 1.0	11.03 ± 0.07
C16:1n-7	7.4 ± 0.0	37.4 ± 0.3	2.86 ± 0.02	8.5 ± 0.1	49.3 ± 0.5	3.41 ± 0.03
C17:0	1.8 ± 0.0	8.9 ± 0.1	0.68 ± 0.01	2.1 ± 0.0	12.5 ± 0.2	0.86 ± 0.01
C18:0	9.6 ± 0.1	48.8 ± 0.9	3.73 ± 0.07	11.0 ± 0.1	64.2 ± 0.6	4.44 ± 0.04
C18:1n-9	20.8 ± 0.0	105.2 ± 0.8	8.04 ± 0.06	15.6 ± 0.1	91.1 ± 0.2	6.30 ± 0.01
C18:2n-6	2.9 ± 0.1	14.7 ± 0.3	1.12 ± 0.02	1.7 ± 0.1	9.8 ± 0.4	0.68 ± 0.03
C18:3n-6	0.4 ± 0.0	2.1 ± 0.1	0.16 ± 0.01	1.1 ± 0.0	6.2 ± 0.3	0.43 ± 0.02
C18:3n-3	2.2 ± 0.0	11.2 ± 0.2	0.86 ± 0.02	0.6 ± 0.0	3.5 ± 0.2	0.24 ± 0.01
C20:2n-6	-	-	-	3.2 ± 0.1	18.5 ± 0.6	1.28 ± 0.04
C20:0	0.4 ± 0.0	2.0 ± 0.0	0.15 ± 0.00	1.0 ± 0.1	6.1 ± 0.4	0.42 ± 0.03
C20:4n-6	2.9 ± 0.0	14.5 ± 0.2	1.11 ± 0.02	-	-	-
C20:5n-3 (EPA)	8.3 ± 0.1	42.1 ± 0.2	3.22 ± 0.02	8.0 ± 0.0	46.9 ± 0.2	3.24 ± 0.01
C22:0	0.4 ± 0.0	2.1 ± 0.1	0.16 ± 0.01	1.2 ± 0.0	6.8 ± 0.3	0.47 ± 0.02
C22:2n-6	0.2 ± 0.0	0.9 ± 0.0	0.07 ± 0.00	1.4 ± 0.1	7.9 ± 0.4	0.55 ± 0.03
C22:6n-3 (DHA)	14.4 ± 0.1	72.7 ± 0.7	5.55 ± 0.05	9.7 ± 0.1	56.3 ± 0.4	3.89 ± 0.03
ΣSFA	38.9 ± 0.1	196.7 ± 1.4	15.03 ± 0.11	50.4 ± 0.1	289.0 ± 2.2	19.97 ± 0.15
ΣMUFA	28.2 ± 0.1	142.7 ± 1.3	10.90 ± 0.10	24.1 ± 0.1	140.4 ± 0.3	9.70 ± 0.02
ΣPUFA	31.3 ± 0.0	158.2 ± 1.0	12.09 ± 0.08	25.6 ± 0.1	149.0 ± 0.5	10.30 ± 0.03
ΣPUFA n-3	24.9 ± 0.1	126.1 ± 0.7	9.63 ± 0.05	18.3 ± 0.1	106.7 ± 0.4	7.37 ± 0.03
ΣPUFA n-6	6.2 ± 0.1	31.3 ± 0.6	2.39 ± 0.05	2.7 ± 0.0	16.0 ± 0.3	1.11 ± 0.02
ΣUFA	59.4 ± 0.2	300.8 ± 2.3	22.98 ± 0.18	49.6 ± 0.1	289.4 ± 0.5	20.00 ± 0.03
EPA + DHA	22.7 ± 0.1	114.8 ± 0.5	8.77 ± 0.04	17.7 ± 0.1	103.2 ± 0.2	7.13 ± 0.01

Values are mean ± SEM (n = 3).

## Data Availability

The data sets used and/or analyzed during the current study are available from the corresponding author upon request.

## Supplementary Materials

The following supporting information can be downloaded at: https://www.mdpi.com/article/10.3390/biology15060495/s1, Figure S1: Lipid Extraction Yield of the Two Seahorse Species (mg/g dry weight); Figure S2: The mixed standard of the FAME GC chromatograms; Figure S3: The relative abundance of triglycerides (TGs) and phospholipids (PLs) in *Hippocampus abdominalis* and *Hippocampus erectus*; Figure S4: Schematic diagram of Total Ion Chromatogram (TIC) of two seahorse species under positive ion mode; Figure S5: Schematic diagram of Total Ion Chromatogram (TIC) of two seahorse species under negative ion mode. Table S1: Fatty acid intra-positional composition in TG of the two *Hippocampus* species; Table S2: Fatty acid intra-positional composition in PC of the two *Hippocampus* species. Table S3: Fatty acid intra-positional composition in PE of the two *Hippocampus* species.

## Abbreviations

The following abbreviations are used in this manuscript:

BisMePA	Bis(methoxyethyl)phosphatidylethanolamine
Cer	Ceramide
CL	Cardiolipin
CITES	Convention on International Trade in Endangered Species of Wild Fauna and Flora
DHA	Docosahexaenoic Acid
dMePE	Dimethylethanolamine Phosphatidylethanolamine
EPA	Eicosapentaenoic Acid
Hex1Cer	Monoglucosylceramide
IACUC	Institutional Animal Care and Use Committee
LPA	Lysophosphatidic Acid
LPC	Lysophosphatidylcholine
LPE	Lysophosphatidylethanolamine
LPI	Lysophosphatidylinositol
LPS	Lysophosphatidylserine
PA	Phosphatidic Acid
PC	Phosphatidylcholine
PE	Phosphatidylethanolamine
PG	Phosphatidylglycerol
PI	Phosphatidylinositol
PL	Phospholipid
SEM	Standard Error of the Mean
SM	Sphingomyelin
TG	Triglyceride
