# Supplementary material for: Lipid Profiles and Fatty Acid Positional Distribution in Two Farmed Seahorse Species by Untargeted Lipidomics and Enzymatic Hydrolysis
Source: Biology (Basel). 2026 Mar 20;15(6):495. doi: 10.3390/biology15060495 (PMC13023979; doi:10.3390/biology15060495)
Supplement: Supplementary file 1 [file biology-15-00495-s001.zip › Figures.pdf]

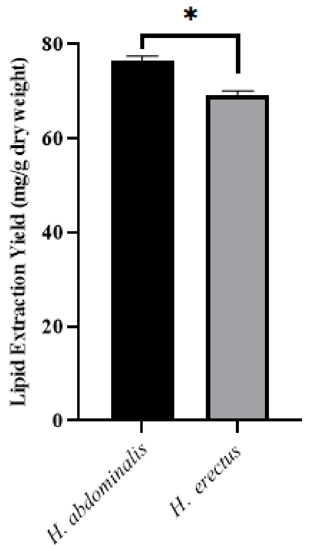

Figure. S1 Lipid Extraction Yield of the Two Seahorse Species (mg/g dry weight).

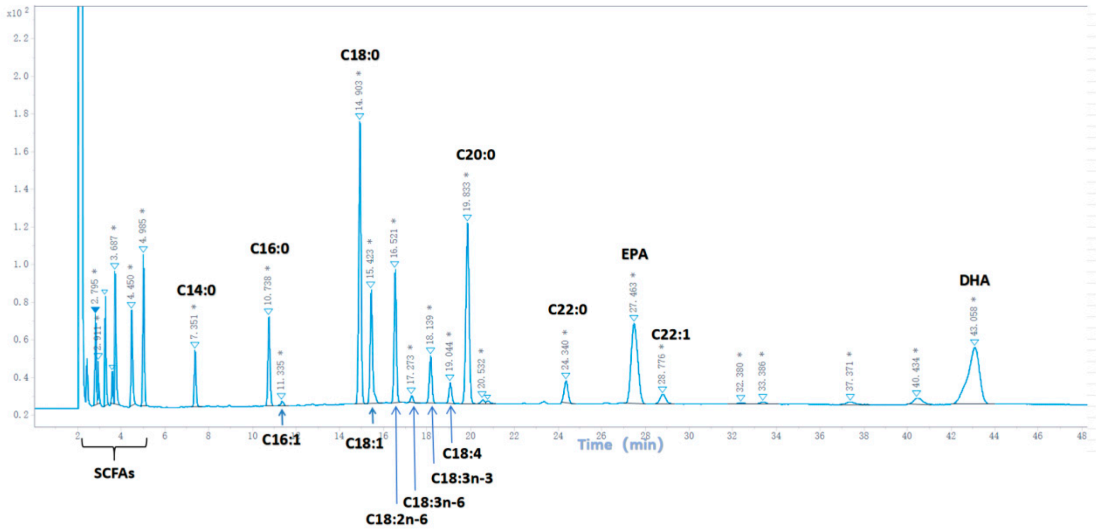

Figure. S2 The mixed standard of the FAME GC chromatograms.

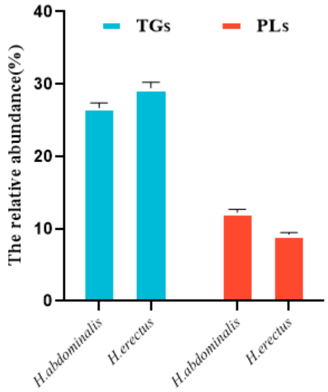

Figure. S3 The relative abundance of triglycerides (TGs) and phospholipids (PLs) in *Hippocampus abdominalis* and *Hippocampus erectus*.

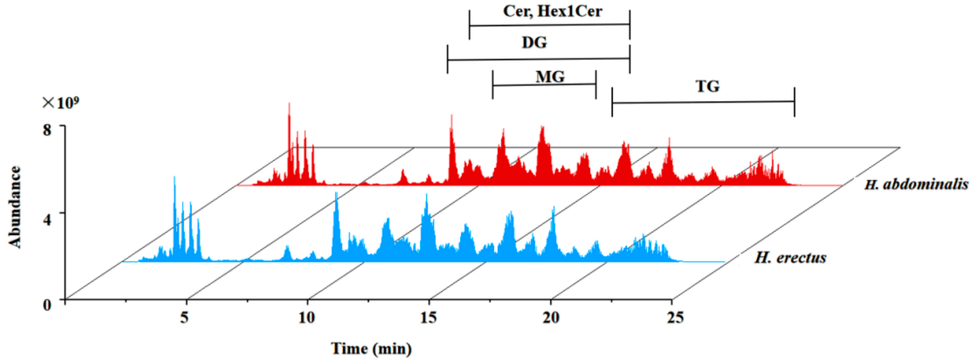

Figure. S4 Schematic diagram of Total Ion Chromatogram (TIC) of two seahorse species under positive ion mode.

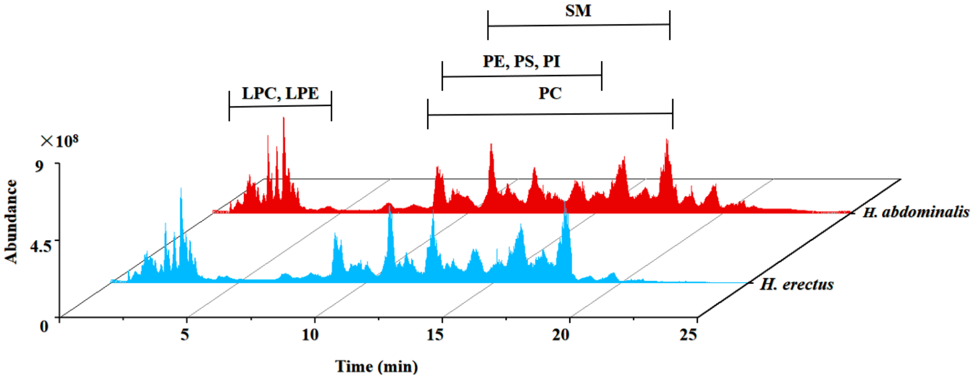

Figure. S5 Schematic diagram of Total Ion Chromatogram (TIC) of two seahorse species under negative ion mode.
