# Supplementary material for: Lipid Profiles and Fatty Acid Positional Distribution in Two Farmed Seahorse Species by Untargeted Lipidomics and Enzymatic Hydrolysis
Source: Biology (Basel). 2026 Mar 20;15(6):495. doi: 10.3390/biology15060495 (PMC13023979; doi:10.3390/biology15060495)
Supplement: Supplementary file 1 [file biology-15-00495-s001.zip › Tables.pdf]

Table S1: Fatty acids intra-positional composition in TG of the two hippocampus species

Fatty acids	*H. abdominalis* Relative abundance (%)			*H. erectus* Relative abundance (%)		
	All	sn-1,3	sn-2	All	sn-1,3	sn-2
C14:0	3.65±0.1	3.77±0.09	3.41±0.41	6.87±0.06	2.46±0.09	15.7±0.31
C16:0	24.57±0.33	26.2±0.21	21.29±0.68	30.78±0.06	31.35±0.16	29.65±0.43
C16:1	7.34±0.12	7.47±0.09	7.06±0.38	7.56±0.07	6.66±0.1	9.38±0.15
C17:0	1.87±0.06	1.84±0.09	1.94±0.03	-	-	-
C18:0	8.23±0.06	6.84±0.09	11.03±0.32	14.34±0.19	16.23±0.06	10.56±0.67
C18:1	23.81±0.1	20.5±0.1	30.44±0.47	19.13±0.12	18.12±0.1	21.14±0.21
C18:2n-6	2.63±0.05	3.21±0.13	1.48±0.35	2.27±0.02	3.2±0.05	0.42±0.12
C18:3n-6	0.74±0.01	0.79±0.03	0.63±0.08	0.94±0.03	1.2±0.05	0.41±0.06
C18:3n-3	1.75±0.07	-	5.24±0.2	0.69±0.04	-	2.07±0.13
C20:2	-	-	-	1.8±0.06	-	5.4±0.18
C20:0	0.93±0.03	1.19±0.03	0.41±0.05	2.22±0.06	2.2±0.09	2.27±0.33
EPA	4.96±0.09	7.19±0.09	0.49±0.24	2.32±0.08	3.28±0.03	0.42±0.2
C22:0	0.4±0.04	0.21±0.06	0.77±0.18	-	-	-
C22:2	1.85±0.05	2.47±0.09	0.62±0.05	1.08±0.05	1.22±0.06	0.8±0.03
DHA	7.65±0.13	7.22±0.07	8.52±0.53	4.4±0.15	6.22±0.17	0.76±0.27
∑SFA	40.05±0.49	40.05±0.34	38.84±0.81	52.24±0.13	52.24±0.22	58.19±0.8
∑MUFA	27.98±0.04	27.98±0.19	37.5±0.45	24.78±0.16	24.78±0.18	30.51±0.21
∑PUFA	20.88±0.39	20.88±0.07	16.97±1.13	15.12±0.25	15.12±0.28	4.88±0.29
∑PUFA n-3	14.41±0.28	14.41±0.09	14.24±0.9	9.5±0.19	9.5±0.15	3.25±0.35
∑PUFA n-6	4±0.06	4±0.16	2.11±0.43	4.4±0.02	4.4±0.1	0.83±0.15
∑UFA	48.85±0.42	48.85±0.25	54.47±1.25	39.9±0.4	39.9±0.44	35.39±0.44
EPA+DHA	14.41±0.22	14.41±0.09	9.01±0.74	9.5±0.15	9.5±0.15	1.18±0.4

Values are mean ± SEM (n = 3).

Table S2: Fatty acids intra-positional composition in PC of the two hippocampus species

Fatty acids	*H. abdominalis* Relative abundance (%)			*H. erectus* Relative abundance (%)		
	All	sn-1	sn-2	All	sn-1	sn-2
C14:0	5.27±0.08	4.27±0.26	6.27±0.13	8.27±0.08	14.48±0.14	2.07±0.03
C16:0	17.49±0.28	0.84±0.37	34.14±0.29	35.46±0.15	27.67±0.24	43.24±0.09
C16:1	3.52±0.06	2.09±0.13	4.95±0.03	-	-	-
C17:0	1.19±0.05	0.71±0.11	1.67±0.03	1.83±0.11	2.93±0.22	0.73±0.02
C18:0	11.92±0.13	10.58±0.12	13.27±0.14	16.53±0.34	23.25±0.71	9.82±0.06
C18:1	14.5±0.1	13.58±0.22	15.41±0.13	11.36±0.18	1.28±0.44	21.43±0.1
C18:2n-6	1.31±0.09	2.47±0.17	0.16±0.04	2.6±0.12	4.84±0.21	0.37±0.06
C18:3n-6	0.25±0.02	0.08±0.01	0.42±0.05	3.9±0.05	7.31±0.09	0.5±0.03
C18:3n-3	0.51±0.05	0.71±0.04	0.31±0.07	2.79±0.09	4.67±0.16	0.9±0.05
C20:2	2.39±0.04	2.37±0.03	2.4±0.05	1.64±0.04	1.17±0.11	2.11±0.05
C20:0	0.45±0.01	0.1±0.04	0.8±0.03	-	-	-
EPA	10.14±0.27	15.47±0.55	4.81±0.07	2.36±0.23	1.2±0.51	3.51±0.09
C22:0	0.64±0.1	1.29±0.19	-	2.47±0.11	4.93±0.21	-
C22:2	1.05±0.02	0.99±0.07	1.11±0.06	-	-	-
DHA	23.92±0.58	44.03±1.05	3.8±0.11	1.25±0.19	1.13±0.38	1.37±0.02
∑SFA	56.14±0.28	17.78±0.45	56.14±0.11	55.85±0.33	73.26±0.66	55.85±0.18
∑MUFA	20.35±0.16	15.67±0.34	20.35±0.16	21.43±0.18	1.28±0.44	21.43±0.1
∑PUFA	13±1.07	63.75±1.81	13±0.34	8.75±0.31	19.14±0.38	8.75±0.15
∑PUFA n-3	8.92±0.89	60.21±1.63	8.92±0.18	5.78±0.1	7±0.08	5.78±0.13
∑PUFA n-6	0.58±0.11	2.55±0.16	0.58±0.07	0.87±0.17	12.14±0.3	0.87±0.1
∑UFA	33.36±1.23	79.42±2.09	33.36±0.33	30.18±0.49	20.42±0.81	30.18±0.09
EPA+DHA	8.6±0.84	59.5±1.59	8.6±0.12	4.88±0.06	2.33±0.13	4.88±0.08

Values are mean ± SEM (n = 3).

Table S3: Fatty acids intra-positional composition in PE of the two hippocampus species

Fatty acids	*H. abdominalis* Relative abundance (%)			*H. erectus* Relative abundance (%)		
	All	sn-1	sn-2	All	sn-1	sn-2
C14:0	0.88±0.08	1.2±0.13	0.57±0.08	5.67±0.1	10.66±0.08	0.69±0.1
C16:0	12.82±0.14	4.72±0.19	20.92±0.14	23.77±0.14	26.14±0.26	21.41±0.14
C16:1	1.88±0.08	2.21±0.07	1.54±0.08	3.14±0	6.29±0.16	-
C17:0	1.51±0	3.03±0.1	-	-	-	-
C18:0	15.99±0.11	15.57±0.28	16.4±0.11	12.45±0.21	12.29±0.49	12.62±0.21
C18:1	12.79±0.11	14.82±0.23	10.77±0.11	20.31±0.09	27.44±0.33	13.18±0.09
C18:2n-6	1.26±0.1	2.22±0.05	0.3±0.1	4.26±0.05	1.63±0.26	6.88±0.05
C18:3n-6	0.44±0.02	0.52±0.07	0.37±0.02	0.31±0.03	0.45±0.06	0.16±0.03
C18:3n-3	0.35±0.06	0.16±0.07	0.54±0.06	0.32±0.13	0.09±0	0.56±0.13
C20:2	0.68±0.02	1.21±0.1	0.16±0.02	2.7±0.35	0.6±0.11	4.8±0.35
C20:0	1.16±0.1	0.97±0.08	1.35±0.1	3.54±0.09	4.92±0.19	2.17±0.09
EPA	7.41±0.13	8.76±0.34	6.07±0.13	4.36±0.13	2.29±0.04	6.44±0.13
C22:0	0.65±0.06	0.18±0.04	1.11±0.06	0.45±0.07	0.3±0.13	0.6±0.07
C22:2	0.84±0	1.69±0.05	-	-	-	-
DHA	26.96±0.05	40.21±0.3	13.72±0.05	1.32±0.06	0.45±0.1	2.19±0.06
∑SFA	40.35±0.36	25.68±0.57	40.35±0.36	37.49±0.42	54.3±0.91	37.49±0.42
∑MUFA	12.31±0.1	17.03±0.17	12.31±0.1	13.18±0.09	33.72±0.34	13.18±0.09
∑PUFA	21.16±0.23	53.55±0.52	21.16±0.23	21.03±0.34	4.92±0.37	21.03±0.34
∑PUFA n-3	20.33±0.16	49.13±0.52	20.33±0.16	9.19±0.06	2.83±0.14	9.19±0.06
∑PUFA n-6	0.67±0.12	2.74±0.05	0.67±0.12	7.04±0.08	2.09±0.25	7.04±0.08
∑UFA	33.47±0.33	70.58±0.36	33.47±0.33	34.2±0.38	38.64±0.31	34.2±0.38
EPA+DHA	19.79±0.17	48.96±0.59	19.79±0.17	8.63±0.19	2.74±0.14	8.63±0.19

Values are mean ± SEM (n = 3).
